# Endotracheal intubation during out‐of‐hospital cardiac arrest: New insights from recent clinical trials

**DOI:** 10.1002/emp2.12003

**Published:** 2019-12-16

**Authors:** Henry E. Wang, Jonathan R. Benger

**Affiliations:** ^1^ Department of Emergency Medicine The University of Texas Health Science Center at Houston Houston Texas; ^2^ Faculty of Health and Applied Sciences University of the West of England Bristol UK

## Abstract

Airway management is an important intervention during resuscitation of out‐of‐hospital cardiac arrest (OHCA). Endotracheal intubation is commonly used by emergency medical services paramedics in the advanced airway management of OHCA, but numerous studies question its safety and effectiveness. Furthermore, there is now increasing use of supraglottic airway devices. In this review, we provide an overview of 3 recent randomized clinical trials of advanced airway management (Pragmatic Airway Resuscitation Trial [PART], AIRWAYS‐2, and Cardiac Arrest Airway Management [CAAM]) and highlight new information that is available to guide OHCA airway management practices.

## 1 INTRODUCTION

Airway management is a core element of resuscitation from cardiopulmonary arrest. In the hospital setting, healthcare practitioners commonly perform the advanced technique of endotracheal intubation (ETI) during cardiac arrest resuscitation in the belief that it provides a direct conduit to the lungs, aids in controlling ventilation and oxygenation, and protects the airway from aspiration. Recognizing that cardiac arrest often occurs outside the hospital, clinical leaders have sought to improve cardiac arrest outcomes by training and equipping paramedics to perform ETI during out‐of‐hospital cardiac arrest (OHCA).^1^, ^2^, ^3^, ^4^, ^5^ In countries with advanced emergency medical services (EMS) systems such as the United States and the United Kingdom, ETI has been the most common approach to advanced airway management in OHCA for over 40 years.

Numerous studies have questioned the role, safety, and effectiveness of ETI in out‐of‐hospital care.^1^ Recent clinical trials—the first ever to randomize advanced airway technique in adult OHCA—have provided new information and perspectives to guide clinical resuscitation practice. In this paper, we summarize the rationale for, results of, and lessons from the Pragmatic Airway Resuscitation Trial (PART), the AIRWAYS‐2 trial, and the Cardiac Arrest Airway Management (CAAM) trial.^6^, ^7^, ^8^

## 2 PITFALLS AND CHALLENGES OF OUT‐OF‐HOSPITAL ENDOTRACHEAL INTUBATION

ETI is a complex procedure entailing over 100 separate manual or cognitive steps.^9^ Studies of paramedic ETI in both OHCA and non‐OHCA cohorts highlight the pitfalls of the intervention. Katz and Falk systematically examined 108 paramedic‐placed endotracheal tubes in patients arriving at the emergency department (ED), finding 25% of the tubes misplaced; two‐thirds were in the esophagus.^10^ First‐pass intubation success is considered an important goal in ETI, but in a series of 1272 OHCA ETI in Pennsylvania, first pass‐success was only 70%.^11^

ETI may also interfere or interact with the multiple simultaneous interventions that occur during resuscitation such as chest compressions, ventilations, defibrillation, vascular access, and drug administration. For example, in OHCA patients treated in Milwaukee, Aufderheide et al. showed that ETI was associated with inadvertent hyperventilation, leading to increased intrathoracic pressure, reduced cardiac preload, and decreased coronary perfusion pressure during OHCA.^12^ In a series of 100 OHCA patients in Pittsburgh, state‐of‐the‐art chest compression detection technology revealed that ETI efforts resulted in at least 2 chest compression interruptions per patient, with the median duration of interruptions totaling over 109 seconds per patient.^13^

In the United States and United Kingdom, opportunities for paramedic acquisition and maintenance of ETI proficiency are limited. Current US national paramedic training standards recommend—but do not require—ETI training on live patients.^14^ Although the operating room offers a venue for learning ETI under controlled conditions, these opportunities are extremely limited, with paramedic students often receiving only 16–32 hours of training in this setting.^15^ In addition, multiple barriers often hinder this experience, including the increasing operating room use of supraglottic airway (SGA) devices, competition from other trainees (eg, medical students, nurse anesthetists, and respiratory therapy students), and increasing medicolegal concerns. In clinical practice, many paramedics perform few ETI procedures. In 2003, Pennsylvania paramedics performed a median of 1 (interquartile range [IQR] 0–2) ETI procedures.^16^ Similar results were found for paramedics in the United Kingdom in 2007.^17^

## 3 SUPRAGLOTTIC AIRWAYS AS AN ALTERNATIVE TO ENDOTRACHEAL INTUBATION

SGA devices include airway devices such as the esophageal‐tracheal combitube (Combitube), laryngeal mask airway (LMA), laryngeal tube (LT), and i‐gel, among others.^18^ Historically, SGA devices were developed for use in the operating room. Prior to the availability of paramedic ETI, there was limited experience with paramedic use of the Combitube and other early SGA devices.^19^, ^20^ With the widespread practice of paramedic ETI, SGA devices were generally relegated to a rescue role after failed intubation efforts.

Pioneering work from Arizona demonstrating the benefit of continuous and minimally interrupted cardiopulmonary resuscitation (CPR) chest compressions resulted in a resurgence of interest in SGA devices.^21^ Given the difficulty of ETI, many EMS agencies resorted to the simpler strategy of SGA insertion to avoid chest compression interruptions during OHCA. Some EMS agencies found that SGA techniques were sufficiently straightforward to allow basic life support rescuers to insert these devices, providing an alternative to bag valve mask (BVM) ventilation.^22^, ^23^

Compared with ETI, SGA devices have a simpler insertion technique and a lower training burden while facilitating ventilation characteristics similar to ETI. Given these factors, one would anticipate better OHCA outcomes with a SGA when compared to ETI insertion. However, analyses of observational data have found better outcomes with ETI than SGA. In an analysis of 10,455 OHCA in the Resuscitation Outcomes Consortium (ROC), ETI was associated with higher odds of a return of spontaneous circulation (ROSC) (odds ratio [OR] 1.78; 95% confidence interval [CI]: 1.54 to 2.04), 24‐hour survival (1.74; 1.49 to 2.04), and hospital discharge with favorable neurologic status (1.40; 1.04 to 1.89) than SGA.^24^ In an analysis of 10,691 OHCA from the Cardiac Arrest Registry to Enhance Survival (CARES), ETI was similarly associated with higher odds of an ROSC (OR 1.35; 95% CI: 1.19 to 1.54), hospital survival (1.41; 1.14 to 1.76), and hospital discharge with favorable neurologic status (1.44; 1.10 to 1.88) than SGA.^25^ In Benoit et al.’s meta‐analysis of 10 observational studies including 34,533 patients with OHCA, ETI was associated with higher odds of hospital discharge with favorable neurologic status (OR 1.33; 95% CI: 1.09 to 1.61) when compared to SGA.^26^

An important limitation of these observational studies is the influence of confounding‐by‐indication. Factors potentially influencing paramedic airway choice may have included the patient's condition, airway anatomy, perceived difficulty of airway management, or practitioner airway skill or comfort with specific airway techniques, among others. Even with the use of advanced analytic techniques such as multivariable adjustment and propensity score matching, it is difficult to fully account for the influence of confounding‐by‐indication, because many confounders may be unknown or unmeasurable.^27^ Thus, randomization is the optimal approach for testing outcomes between different OHCA airway management techniques.

## 4 CLINICAL TRIALS OF ENDOTRACHEAL INTUBATION IN OUT‐OF‐HOSPITAL CARDIAC ARREST

Motivated by the prominence of airway management during OHCA, the uncertain safety of ETI, and the unclear effectiveness of SGA strategies, 3 recent multicenter clinical trials (the first ever of airway management techniques in adult OHCA) tested the effectiveness of ETI in the resuscitation of OHCA patients (Table 1).

**Table 1:** Characteristics of the Pragmatic Airway Resuscitation Trial (PART), the AIRWAYS‐2 trial, and the Cardiac Arrest Airway Management (CAAM) trial

Characteristic	PART	AIRWAYS‐2	CAAM
Setting	United States	United Kingdom	France, Belgium
Patients	Adult (≥18 years) OHCA requiring BVM or advanced airway management	All adult (≥18 years) OHCA	All adult (≥18 years) OHCA
Comparisons	Laryngeal tube versus endotracheal intubation	i‐gel versus endotracheal intubation	Bag‐valve‐mask ventilation versus endotracheal intubation
Practitioners	Paramedics, select emergency medical technicians	Paramedics	Physicians, nurses, paramedics
Total enrollment	3004	9296	2043
Design	Superiority	Superiority	Non‐inferiority
Method of randomization	Cluster randomization with cross‐over. Randomization units defined by EMS agencies	Cluster randomization without cross‐over. Randomized by individual paramedic	Randomization by patient—assignment determined by sealed opaque envelopes
Primary outcome	72‐hour survival	Hospital survival with favorable functional status	28‐day survival with favorable neurologic status
Patients not receiving any of study interventions	∼12%^a^	∼18%^b^	∼0.7%^c^
Primary finding	LT superior to ETI (72‐hour survival LT 18.3% versus ETI 15.4%, difference 2.9% (95% CI: 0.2–5.6), *P* = 0.04	No difference between i‐gel and ETI (hospital survival with favorable functional outcome i‐gel 6.4% versus ETI 6.8%, OR 0.92% (95% CI: 0.77–1.09), *P* = 0.33)	Inconclusive (28‐day survival with favorable neurologic status BVM 4.3% versus ETI 4.2%, difference 0.11% (one‐sided 97.5% CI: −1.64 to 0), non‐inferiority *P* = 0.11)
Important secondary findings	Shorter EMS‐to‐airway time in LT than TI. Low airway insertion success rate in the ETI arm (51.6%). Allocation imbalance in select randomization clusters.	Differential use of advanced airway management between groups. Superior initial ventilation success with i‐gel. No difference in regurgitation and aspiration between randomization groups.	BVM associated with more difficult ventilation and higher aspiration.

a Patients who did not receive ETI or LT.

b Patients who did not receive ETI or i‐gel.

c Patients who did not receive ETI or BVM.

### 4.1 The Pragmatic Airway Resuscitation Trial

The Pragmatic Airway Resuscitation Trial (PART) involved 27 US advanced life support EMS agencies from the Birmingham (Alabama), Dallas‐Fort Worth, Milwaukee, Pittsburgh, and Portland (Oregon) sites of the ROC.^6^ The trial included adult OHCA patients who required BVM ventilation or advanced airway insertion. The trial tested 2 airway strategies: (1) initial airway management with the LT, or (2) initial airway management with ETI. EMS personnel were permitted to use any available airway technique to rescue failed initial airway efforts. Basic life support personnel that possessed existing LT insertion skills performed LT when the agency was assigned to the LT arm and BVM ventilation when the agency was assigned to the ETI arm. The 27 EMS agencies were organized into 13 randomization clusters that alternated airway assignments at predefined 3‐ to 5‐month intervals. All other aspects of OHCA care followed local protocol.

The primary outcome of PART was 72‐hour survival, an endpoint that was selected because of the limited funding available for the study and the lower required sample size. This time interval also accounted for the common use of therapeutic hypothermia, percutaneous coronary intervention, and delayed withdrawal of care in current post‐OHCA management in the United States. The trial also assessed survival to hospital discharge and hospital survival with good functional status (Modified Rankin Scale [MRS] ≤3) as secondary outcomes. Patient enrollment occurred between December 2015 and October 2017.

Of 3004 enrolled patients, 3000 were included in the analysis. PART found that the strategy of initial LT resulted in higher 72‐hour survival than a strategy of initial ETI; 18.3% versus 15.4%, difference 2.9% (95% CI: 0.2–5.6), *P* = 0.04. Although the study was powered to detect differences in 72‐hour survival, the observed treatment effects persisted for survival to hospital discharge (LT 10.8% versus ETI 8.1%, difference 2.7% (95% CI: 0.6%–4.8%), *P* = 0.01) and hospital survival with favorable neurologic status (LT 7.1% versus ETI 5.0%, difference 2.1% (95% CI: 0.3%–3.8%), *P* = 0.02. These results suggested better overall OHCA outcomes with a strategy of initial LT than initial ETI.

There were several important secondary findings and limitations in PART. Elapsed time from EMS arrival to airway start was almost 3 minutes shorter with LT than ETI (median 9.8 versus 12.5 minutes), supporting the hypothesis that LT is more efficient than TI. The intubation success rate in the ETI arm was 51.6%, a figure below that reported by meta‐analyses (91.2%).^28^ However, the majority of these cases were successfully rescued by LT, resulting in an overall airway success rate of 91.5% in the ETI arm. Although the exact reasons for the lower ETI success rate were not clear, the observations were consistent with the common practice of limiting futile ETI efforts and favoring early rescue LT use. There were also imbalances in treatment allocation within select randomization clusters; post hoc multivariable adjustment to account for these imbalances attenuated some of observed associations between LT and OHCA outcomes.

### 4.2 AIRWAYS‐2 trial

Conducted in the United Kingdom, the AIRWAYS‐2 trial included 4 advanced life support EMS agencies serving a population of over 21 million people.^7^ In contrast to PART, AIRWAYS‐2 included all adult non‐traumatic OHCA patients; not just those requiring BVM or advanced airway management. The trial tested initial airway management using (1) i‐gel, or (2) ETI. Unlike PART, AIRWAYS‐2 cluster randomized by paramedic. The study solicited paramedic participation in the trial, randomizing each of the 1523 participating paramedics to either the i‐gel or ETI strategy for the duration of the trial. Paramedics did not cross over to the alternate treatment arm. All other aspects of OHCA care and subsequent in‐hospital management followed local protocols. The trial was powered to detect differences in hospital survival with favorable functional outcome (MRS score ≤3; a secondary outcome in PART). Patient enrollment occurred between June 2015 and August 2017.

AIRWAYS‐2 enrolled a total of 9296 patients. The trial observed no difference in the primary outcome of hospital survival with favorable functional outcome; i‐gel 6.4% versus ETI 6.8%, OR 0.92% (95% CI: 0.77–1.09), *P* = 0.33. The study similarly observed no difference in survival (i‐gel 8.0% versus ETI 8.4%). Overall, these results suggested no differences in outcome between the i‐gel and ETI treatment groups.

An important limitation of AIRWAYS‐2 was that approximately 18% of patients (22% of ETI, 15% of i‐gel) did not receive advanced airway management (neither i‐gel nor ETI attempts). This figure was higher than the 12% BVM‐only rate observed in PART because AIRWAYS‐2 included all OHCA, not just those requiring BVM or advanced airway management. The possibility that advanced airway management techniques would not be used was a recognized possibility in both studies, reflecting clinical scenarios with early ED arrival, early ROSC, or where EMS personnel felt that ventilation was adequate with BVM alone. However, to avoid the possibility of selection bias by unblinded paramedics, AIRWAYS‐2 included all OHCA patients, whereas PART included only those patients who received BVM or were anticipated to require advanced airway management. This may explain the difference in the use of advanced airway management between the 2 studies.

In AIRWAYS‐2, the investigators conducted a pre‐specified sensitivity analysis limited to the 7576 patients who received i‐gel or ETI, finding better hospital survival with favorable functional outcome in the i‐gel than the ETI arm (3.9% versus 2.6%; difference 1.4%, 95% CI: 0.5%–2.2%). This finding is limited by its exploratory nature and is likely to be explained by selection bias given the differential use of advanced airway techniques between the 2 randomization groups. However, initial ventilation success (up to 2 attempts) was significantly better in the i‐gel group (87% versus 79.0%; difference 8.3%, 95% CI: 6.3%–10.2%).

### 4.3 The Cardiac Arrest Airway Management trial

Although the PART and AIRWAYS‐2 trials compared ETI with SGA insertion, a more fundamental question is whether ETI demonstrates any benefit over the more basic technique of BVM ventilation. Using data from approximately 650,000 patients with OHCA recorded in the all‐Japan Utstein registry, Hasegawa et al. found that advanced airway management was associated with lower 30‐day favorable neurologic status than BVM ventilation (1.1% versus 2.9%; adjusted OR 0.38; 95% CI: 0.37–0.40).^29^ The Cardiac Arrest Airway Management (CAAM) trial compared the effects of ETI and BVM ventilation on OHCA outcomes.^8^

CAAM included 20 EMS ambulance base stations in France and Belgium, each with one or more mobile intensive care Service d'Aide Médicale Urgente (SAMU) units. In contrast to the United States and the United Kingdom, the French and Belgium SAMU units are typically staffed by an anesthetist or emergency physician, a nurse, and an ambulance driver. Adult patients with OHCA were randomized to receive either ETI or BVM ventilation, with randomization assignment determined on an individual basis by sealed opaque envelopes. All patients achieving ROSC subsequently received ETI. The primary outcome was 28‐day survival with favorable neurologic status (Glasgow‐Pittsburgh Cerebral Performance Category ≤2). Unlike PART and AIRWAYS‐2, CAAM used a non‐inferiority design, with the non‐inferiority margin set at 1% (non‐inferiority present if BVM outcome no more than 1% lower than ETI). The trial enrolled subjects between March 2015 and January 2017.

CAAM enrolled a total of 2000 patients. BVM showed slightly higher 28‐day favorable neurologic status than ETI; 4.3% versus 4.2%; difference 0.11% (one‐sided 97.5% CI: −1.64% to 0). However, the study could not demonstrate the non‐inferiority of BVM compared with ETI (non‐inferiority *P* = 0.11). There were no discernable differences in survival to hospital admission or 28‐day survival.

There were 2 notable secondary findings in CAAM. With intubation difficulty defined as an Intubation Difficulty Scale >5 and BVM difficulty defined by Han scale >2, EMS physicians reported greater airway management difficulty in the BVM than the ETI group; 18.1% versus 13.4%, difference 4.6% (95% CI: 2.8%–6.4%), *P* < 0.001.^30^ Reported regurgitation of gastric contents was higher in the BVM than the ETI group; 15.2% versus 7.5%, difference 7.7% (95% CI: 4.9%–10.4%), *P* < 0.001. It is also worth noting that SAMU arrival may occur relatively late in cardiac arrest, limiting the scope for these airway interventions to influence patient outcome.

## 5 IMPLICATIONS FOR CLINICAL PRACTICE

The interpretation and clinical application of the findings of PART, AIRWAYS‐2, and CAAM present important challenges. PART suggests that the use of an SGA device (in this case, LT) could be the strategy of choice for all EMS providers in the resuscitation of patients with OHCA. The larger AIRWAYS‐2 trial partially supports this interpretation, with the primary outcome suggesting that ETI offers no clinical advantage over the i‐gel SGA. The CAAM trial does not suggest a benefit for ETI over BVM. Therefore, none of these 3 randomized trials—the best evidence available to date regarding advanced airway management in adult OHCA—indicate clinical advantages for ETI over SGA or BVM in adult OHCA. Given these findings, the complexity of ETI and the significant resource investment needed for paramedics to attain and maintain ETI proficiency, one might expect clinical practice to shift from ETI to primary SGA use in OHCA resuscitation.

However, some EMS medical directors and clinicians may take a contrasting view. Critics highlight the low ETI success rate of PART, noting that these figures do not reflect typical paramedic ETI practice, and suggest that OHCA outcomes may have been different with better paramedic ETI skills. These individuals may also view the absence of a difference between i‐gel and ETI in AIRWAYS‐2 as evidence that “ETI is not harmful” compared with the i‐gel SGA. Another consideration is that over two‐thirds of ETI by EMS personnel occurs in OHCA; replacement of OHCA ETI with SGA would significantly diminish paramedic clinical ETI experience, potentially undermining the ability to perform ETI in other patients and to undertake laryngoscopy for other reasons (eg, removal of a foreign body in the upper airway).^31^ There will also be some adult patients in OHCA for whom neither BVM nor a SGA will achieve effective ventilation. These collective factors may lead some EMS agencies—especially those with strong ETI training resources and a workforce that favors ETI—to maintain the practice of ETI in OHCA resuscitation.

Although some experts have proposed BVM as a preferred approach over both ETI and SGA, CAAM found higher rates of airway management difficulty and regurgitation with BVM, diminishing its appeal in clinical practice. PART and AIRWAYS‐2 did not incorporate a BVM‐only arm because of paramedic reluctance to use BVM alone; EMS personnel viewed BVM as difficult and impractical in the setting of a patient with OHCA, distracting providers from other resuscitation priorities. The findings of CAAM support these perspectives. Although CAAM's non‐inferiority design led to an uninterpretable result, a repeat of the trial is unlikely given its important secondary findings.

## 6 UNANSWERED QUESTIONS

Despite the scale of the 3 trials, important scientific questions remain. PART, AIRWAYS‐2, and CAAM evaluated different airway devices; there has been only 1 direct randomized comparison between different SGA devices (eg, LT, i‐gel, or LMA), and none between SGA and BVM in randomized clinical trials enrolling adult OHCA patients.^32^ There is also limited information regarding the influence of airway management technique on chest compression continuity. AIRWAYS‐2 and CAAM collected CPR process data on very few patients (n = 66 and 115, respectively), and PART lacked sufficient resources to support CPR process data collection and analysis.

Although widely used in clinical practice, there are relatively few large reports of adverse events associated with out‐of‐hospital SGA use.^33^ In a porcine model of OHCA, Segal et al. showed that advanced airway devices—including ET tubes, LT, LMA, and Combitube—resulted in a reduction of carotid blood flow; these findings are potentially important but have yet to be confirmed in human cardiac arrests.^34^ If SGA devices are broadly adopted into practice, continued surveillance for adverse events will be essential.

Although critics suspect the low ETI success rate of PART may have reduced survival in the ETI arm, this mediating relationship has yet to be verified. Portable video laryngoscopy (VL) is now broadly available in the out‐of‐hospital setting and could ease intubation efforts. Widespread availability of VL could alter the perceived tradeoffs between ETI and SGA, but any impact on OHCA outcomes would merit formal evaluation.^32^ The technique of passive ventilation (high flow oxygen by face‐mask only, without BVM or advanced airway) was demonstrated in Arizona's implementation of minimally interrupted CPR; while potentially circumventing all issues with advanced airway management, this technique has yet to be tested in a randomized fashion.^21^

Whereas PART, AIRWAYS‐2, and CAAM focused on adult OHCA, other important patient groups include those with trauma (both traumatic brain injury and hemorrhagic shock) and medical non‐arrest conditions such as acute pulmonary edema, seizures, and drug overdoses. The multicenter Prehospital Airway Control in Trauma (PACT) trial of the Linking Investigations in Trauma and Emergency Services (LITES) network will soon compare ETI versus i‐gel insertion in major trauma.^35^ Furthermore, PART, AIRWAYS‐2, and CAAM all focused on adults. Gausche et al.’s landmark trial of ETI versus BVM in children occurred 20 years ago, combined all disease conditions, and did not include SGA devices, which are now available in pediatric sizes.^36^ A contemporary pediatric airway management trial should compare the effectiveness of ETI, SGA, and BVM, and should stratify by medical condition (eg, cardiac arrest, medical non‐arrest, trauma).

In conclusion, airway management is an essential component of OHCA resuscitation. The PART, AIRWAYS‐2, and CAAM trials provide vital new information to inform the practice of airway management in patients with OHCA, but important questions remain.

## AUTHOR CONTRIBUTIONS

HEW conceived the paper and drafted the manuscript. HEW and JRB contributed critical review and revision. HEW assumes overall responsibility for the paper.

## CONFLICTS OF INTEREST

The authors have no conflict of interest to disclose.
